# Peer-Delivered Linkage Case Management and Same-Day ART Initiation for Men and Young Persons with HIV Infection — Eswatini, 2015–2017

**DOI:** 10.15585/mmwr.mm6723a3

**Published:** 2018-06-15

**Authors:** Duncan MacKellar, Daniel Williams, Bonsile Bhembe, Makhosazana Dlamini, Johnita Byrd, Lenhle Dube, Sikhathele Mazibuko, Trong Ao, Ishani Pathmanathan, Andrew F. Auld, Pamela Faura, Nomthandazo Lukhele, Caroline Ryan

**Affiliations:** ^1^Division of Global HIV and TB, Center for Global Health, CDC; ^2^Population Services International Country Program, Mbabane, Eswatini; ^3^National AIDS Programme, Eswatini Ministry of Health, Mbabane, Eswatini; ^4^CDC Country Office, Mbabane, Eswatini.

To achieve epidemic control of human immunodeficiency virus (HIV) infection, sub-Saharan African countries are striving to diagnose 90% of HIV infections, initiate and retain 90% of HIV-diagnosed persons on antiretroviral therapy (ART), and achieve viral load suppression* for 90% of ART recipients (90-90-90) (*1*). In Eswatini (formerly Swaziland), the country with the world’s highest estimated HIV prevalence (27.2%), achieving 90-90-90 depends upon improving access to early ART for men and young adults with HIV infection, two groups with low ART coverage (*1*–*3*). Although community-based strategies test many men and young adults with HIV infection in Eswatini, fewer than one third of all persons who test positive in community settings enroll in HIV care within 6 months of diagnosis after receiving standard referral services (*4*,*5*). To evaluate the effectiveness of peer-delivered linkage case management^†^ in improving early ART initiation for persons with HIV infection diagnosed in community settings in Eswatini, CDC analyzed data on 651 participants in CommLink, a community-based, mobile HIV-testing, point-of-diagnosis HIV care, and peer-delivered linkage case management demonstration project, and found that after diagnosis, 635 (98%) enrolled in care within a median of 5 days (interquartile range [IQR] = 2–8 days), and 541 (83%) initiated ART within a median of 6 days (IQR = 2–14 days), including 402 (74%) on the day of their first clinic visit (same-day ART). After expanding ART eligibility to all persons with HIV infection on October 1, 2016, 96% of 225 CommLink clients initiated ART, including 87% at their first clinic visit. Compared with women and adult clients aged ≥30 years, similar high proportions of men and persons aged 15–29 years enrolled in HIV care and received same-day ART. To help achieve 90-90-90 by 2020, the United States President’s Emergency Plan for AIDS Relief (PEPFAR) is supporting the national scale-up of CommLink in Eswatini and recommending peer-delivered linkage case management as a potential strategy for countries to achieve >90% early enrollment in care and ART initiation after diagnosis of HIV infection (*6*).

CommLink was implemented by two outreach teams, each operating with a van in rural and urban catchment areas in the Hhohho and Manzini regions of Eswatini during June 2015–March 2017 (Figure 1). Each team included two or three HIV-test counselors, three HIV-positive, ART-adherent expert client (peer) counselors, and a nurse. HIV testing was offered to persons encountered at homesteads, worksites, bars, and high-traffic urban locations (e.g., near markets and bus stops). Clients who tested positive and had not received HIV care in the past 90 days were offered point-of-diagnosis HIV care and linkage case management. In modified vans (mobile units) parked at test locations, CommLink nurses provided physical and psychosocial assessment, clinical staging, CD4 count, syndromic treatment for sexually transmitted infections, and a 7-day course of cotrimoxazole (Figure 2). Medical files completed by CommLink nurses were transferred within 48 hours to clinics, health centers, and other referral facilities where clients could receive ART (Figure 1).

**FIGURE 1:**
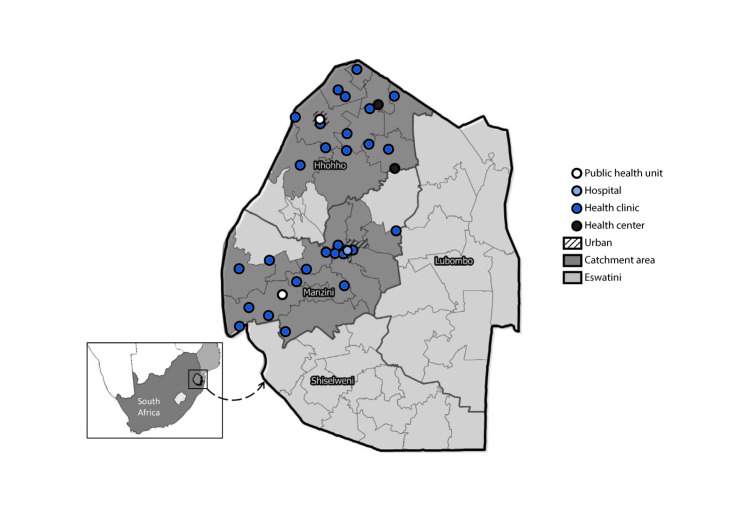
CommLink* catchment areas and referral HIV-care facilities — Eswatini,^†^ June 2015–March 2017 **Abbreviation:** HIV = human immunodeficiency virus. * CommLink is a community-based, mobile HIV-testing, point-of-diagnosis HIV care, and peer-delivered linkage case management demonstration project. ^†^ Formerly Swaziland.

**FIGURE 2:**
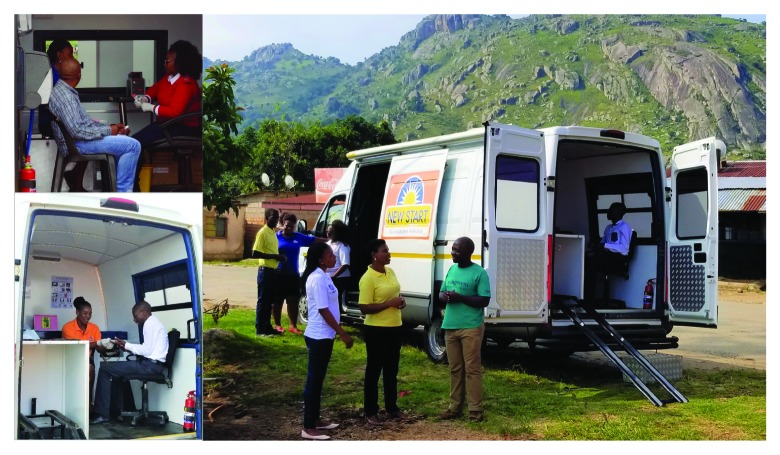
CommLink* outreach testing with point-of-diagnosis HIV-care services — Eswatini,^†^ June 2015–March 2017 **Abbreviation:** HIV = human immunodeficiency virus. * CommLink is a community-based, mobile HIV-testing, point-of-diagnosis HIV care, and peer-delivered linkage case management demonstration project. ^†^ Formerly Swaziland.

Peer counselors provided linkage case management for consenting clients from the time of diagnosis through at least the first return visit for facility-based care after ART initiation. For ART-eligible clients who did not initiate ART or return for their first refill of antiretroviral medication, linkage case management services continued for up to 90 days. Linkage case management comprises the package of services recommended by CDC^§^ and the World Health Organization (WHO), including 1) peer-delivered counseling and psychosocial support; 2) treatment navigation at referral facilities (escorting to or meeting clients at the referral facility at least once, providing psychosocial support [for the duration of the first clinic visit], and explaining the content, sequence, and locations of HIV clinical, laboratory, and pharmaceutical services); 3) weekly telephone calls and appointment reminders; and 4) two follow-up face-to-face counseling sessions on disclosing HIV status to and HIV testing of partners and family members and on identifying and resolving real and perceived barriers to HIV care (*1*,*7*).

National guidelines for ART eligibility based on CD4 count were expanded twice during the project, resulting in the following three ART-eligibility periods: 1) June 2015–November 2015 (CD4 count <350/*µ*L); 2) December 2015–September 2016 (CD4 count <500/*µ*L); and 3) October 2016–March 2017 (any CD4 count, Test and Start^¶^). At referral facilities, same-day ART patients received a 14-day supply of antiretroviral medications and were instructed to return in 2 weeks to receive their baseline laboratory test results and their first 30-day antiretroviral refill. Information on receipt of facility-based clinical services, including ART, and associated dates of service were abstracted from patient health care cards.

Among 909 persons who tested HIV-positive during CommLink outreach events, 21 (2%) left the event before eligibility screening, and 163 (18%) were either currently in HIV care (90), requested a referral to a facility outside of the catchment area (33), or were ineligible for linkage case management for other reasons, such as residence in another region or country (40). Among 725 eligible persons, 19 (2.6%) were aged <15 years and were excluded from analyses. Of 706 eligible persons aged ≥15 years, 651 (92%) participated in linkage case management and received services for a median of 42 days (IQR = 24–66 days).

Excluding weekly telephone contacts, >90% of clients in all demographic and diagnostic subgroups, including men, persons aged 15–29 years, participants from urban outreach events, and participants who had counselors of a different gender, received linkage case management services (Table). Although proportionally fewer male than female counselors documented weekly telephone contacts with their clients, male counselors contacted 236 (99.6%) of their 237 clients by phone at least three times.

**TABLE:** Use of CommLink* services and enrollment in HIV care and same-day ART initiation outcomes, by client and project characteristics — Eswatini,^†^ June 2015–March 2017^§^

Characteristic	CommLink clients no. (%)	Mobile HIV care^¶^ no. (%)	Treatment navigation** no. (%)	Weekly telephone contact^††^ no. (%)	Counseling sessions^§§^ no. (%)	Enrolled in HIV care^¶¶^ no. (%)	Initiated on ART*** no. (%)	Same-day ART^†††^ no. (%)	Same-day ART returned^§§§^ no. (%)
**Total**	**651 (100)**	**629 (97)**	**621 (95)**	**553 (85)**	**608 (93)**	**635 (98)**	**541 (83)**	**402 (74)**	**379 (94)**
**Sex**									
Male	411 (63)	397 (97)	393 (96)	351 (85)	383 (93)	399 (97)	346 (84)	251 (73)	234 (93)
Female	240 (37)	232 (97)	228 (95)	202 (84)	225 (94)	236 (98)	195 (81)	151 (77)	145 (96)
**Age group (yrs)**									
15–24	91 (14)	86 (95)	84 (92)	74 (81)	84 (92)	89 (98)	72 (79)	50 (69)	48 (96)
25–29	149 (23)	142 (95)	143 (96)	125 (84)	140 (94)	143 (96)	118 (79)	89 (75)	84 (94)
30–34	144 (22)	141 (98)	135 (94)	125 (87)	135 (94)	139 (97)	118 (82)	91 (77)	88 (97)
35–44	169 (26)	166 (98)	163 (96)	144 (85)	160 (95)	167 (99)	145 (86)	110 (76)	100 (91)
≥45	98 (15)	94 (96)	96 (98)	85 (87)	89 (91)	97 (99)	88 (90)	62 (70)	59 (95)
**HIV diagnostic status**									
New	443 (68)	426 (96)	420 (95)	365 (82)	414 (93)	429 (97)	361 (81)	261 (72)	246 (94)
Prior, out-of-care^¶¶¶^	208 (32)	203 (98)	201 (97)	188 (90)	194 (93)	206 (99)	180 (87)	141 (78)	133 (94)
ART-eligibility period****									
Jun 2015–Nov 2015 (CD4 <350/*µ*L)	137 (21)	123 (90)	119 (87)	102 (74)	115 (84)	127 (93)	90 (66)	56 (62)	53 (95)
Dec 2015–Sep 2016 (CD4 ≤500/*µ*L)	289 (44)	285 (99)	281 (97)	248 (86)	273 (94)	285 (99)	234 (81)	158 (68)	148 (94)
Oct 2016–Mar 2017 (Test and Start)	225 (35)	221 (98)	221 (98)	203 (90)	220 (98)	223 (99)	217 (96)	188 (87)	178 (95)
**Outreach setting**									
Urban	346 (53)	340 (98)	329 (95)	289 (84)	323 (93)	337 (97)	275 (79)	186 (68)	176 (95)
Rural	305 (47)	289 (95)	292 (96)	264 (87)	285 (93)	298 (98)	266 (87)	216 (81)	203 (94)
**Counselor-client dyads**									
Female-male	261 (40)	250 (96)	255 (98)	235 (90)	244 (93)	256 (98)	219 (84)	159 (73)	149 (94)
Female-female	153 (24)	146 (95)	145 (95)	139 (91)	145 (95)	150 (98)	121 (79)	89 (74)	85 (96)
Male-female	87 (13)	86 (99)	83 (95)	63 (72)	80 (92)	86 (99)	74 (85)	62 (84)	60 (97)
Male-male	150 (23)	147 (98)	138 (92)	116 (77)	139 (93)	143 (95)	127 (85)	92 (72)	85 (92)

**Abbreviations:** ART = antiretroviral therapy; HIV = human immunodeficiency virus; IQR = interquartile range; LCM = linkage case management.

* CommLink is a community-based, mobile HIV-testing, point-of-diagnosis HIV care, and peer-delivered LCM demonstration project.

^†^ Formerly Swaziland.

^§^ Duration of CommLink services: median interval = 42 days, IQR = 24–66 days.

^¶^ Includes clinical assessment, CD4+ T-cell count/µL (CD4 count) testing, syndromic treatment for sexually transmitted infections, and cotrimoxazole preventive therapy provided by CommLink nurses at HIV diagnosis.

** Client accompanied by CommLink peer counselor for the duration of at least the first HIV-care facility visit and received psychosocial support and informational counseling on the content and location of HIV clinical, laboratory, and pharmaceutical services.

^††^ Client spoke with peer counselor, on average, at least once per week for the duration of CommLink services.

^§§^ Client received initial and at least two follow-up face-to-face counseling sessions focused on the importance of early enrollment in HIV care and ART, disclosure to and HIV testing of partners and family members, and identifying and resolving real and perceived barriers to enrollment or retention in HIV care.

^¶¶^ Documentation on patient’s health care card of receipt of HIV care services at least once at a standing fixed facility (clinic, health center, or hospital). Median interval from HIV diagnosis to enrollment in HIV care = 5 days (IQR = 2–8).

*** ART initiation among patients who met national eligibility guidelines is not provided because of observed variation in ART initiation practices across facilities attributed to 1) a Test and Start study conducted at multiple northern facilities and 2) facility-specific interpretation of expanding national treatment guidelines. Percentages are of all CommLink clients. Median interval from HIV diagnosis to ART initiation = 6 days (IQR = 2–14).

^†††^ Initiated during the first facility visit. Typical practice is to provide a 14-day starter pack of antiretroviral medication. Percentages are of patients initiated on ART.

^§§§^ Returned for HIV care at the facility at least once after same-day ART initiation; median interval from ART initiation to return visit = 14 days (IQR = 14–15). The return visit was typically to receive baseline test results and the first 30-day antiretroviral medication refill. Percentages are of patients initiated on ART.

^¶¶¶^ Client reported a prior HIV diagnosis but not having received HIV care in >90 days.

**** Changes in national ART polices based on CD4 count; Test and Start = ART for all HIV-infected persons regardless of CD4 count.

From the date of diagnosis, 635 (98%) clients received HIV care at least once at a referral facility within a median of 5 days (IQR = 2–8 days), and 541 (83%) initiated ART within a median of 6 days (IQR = 2–14 days), including 402 (74%) on the day of their first clinic visit (Table). As ART eligibility increased from a required CD4 count <350/*µ*L to Test and Start, the percentage of all clients initiated on ART increased from 66% to 96%, the percentage of clients initiated on ART who received same day ART increased from 62% to 87%, and, among 361 clients with newly diagnosed HIV infection, the median CD4 count at ART initiation increased from 313/*µ*L (IQR = 203/*µ*L–422/*µ*L) to 454/*µ*L (IQR = 264/*µ*L–598/*µ*L). Among 402 clients who initiated ART on the day of their first clinic visit, 379 (94%) returned to the facility at least once after ART initiation within a median of 14 days (IQR = 14–15 days).

Nearly all clients enrolled in facility-based HIV care, including men (97%), persons aged 15–29 years (97%), participants from urban (97%) and rural (98%) outreach events, and participants with counselors of the same or different gender (95%–99%). Compared with women and adult clients aged ≥30 years, similar high proportions of men and clients aged 15–29 years received same-day ART and returned to care after same-day ART initiation (Table).

## Discussion

Among 651 persons with HIV infection participating in CommLink, a PEPFAR-funded, community-based, mobile HIV-testing, point-of-diagnosis HIV care, and peer-delivered linkage case management demonstration project in Eswatini, nearly all received recommended linkage services, and most enrolled in facility-based HIV care and initiated ART within a few days of the start of these services. During Test and Start, nearly all (96%) CommLink clients initiated ART, most (87%) on the day of their first clinic visit. CommLink findings of near universal early enrollment in HIV care and ART initiation stand in contrast to other studies in Eswatini and elsewhere in sub-Saharan Africa suggesting that only 26%–37% of persons provided standard referral services after HIV diagnosis in community settings enroll early in HIV care, and that many, particularly young adults, delay their enrollment in HIV care for years (*4*,*5*,*8*,*9*).

Early ART initiation after diagnosis is essential to prevent HIV-associated morbidity and mortality and HIV transmission to sexual partners and offspring (*10*). As ART guidelines were expanded in Eswatini, both the percentage of CommLink clients initiated on ART and the median CD4 count at ART initiation increased, suggesting programs that integrate community-based HIV testing with recommended linkage and same-day Test and Start services can help reduce late ART initiation and prevalence of advanced HIV disease (*10*).

As recommended by CDC and WHO, CommLink peer-delivered linkage case management services are initiated for all consenting clients at the point of diagnosis (*1*,*7*). Reactive linkage programs (those that require either referral forms or documentation of missed appointments to initiate follow-up) might miss important opportunities to provide timely and effective linkage services (*5*,*9*). As a proactive program, CommLink peer counselors initiate services at the time of diagnosis to build rapport, assess and understand individual circumstances, and use their personal experiences living with HIV infection to help clients cope with their diagnosis, correct misperceptions about HIV and ART, assess and mitigate barriers to HIV care, and ensure that all participants understand how to navigate HIV care. These services might be particularly helpful to groups at high risk for delayed enrollment in HIV care, such as men and young adults.

The findings in this report are subject to at least three limitations. First, clinical outcomes on patient health care cards are subject to documentation and data-abstraction errors. Senior investigator audits of 165 (26%) medical charts of clients enrolled at 12 facilities, however, found that all abstracted enrollment, ART-initiation, and return-visit data were complete and accurate. Second, because cases were closed within 90 days, retention in HIV care among CommLink clients is unknown. However, nearly all same-day ART patients returned to care at least once, suggesting that retention outcomes might be similar to other ART patient cohorts (*1*,*8*). Finally, although CommLink enrollment-in-care findings far exceed those of historical community-based cohorts in Eswatini and elsewhere in sub-Saharan Africa, some of the differences might also be attributed to improvements in decentralized services, Test and Start policies, and HIV care–seeking societal norms (*1*,*8*). However, even when all persons who receive a diagnosis of HIV infection in community settings in sub-Saharan Africa are informed they will receive ART, few (37%) enroll early in care and initiate ART when provided standard referral services alone (*9*).

As a demonstration project providing the package of linkage services that are recommended by CDC and WHO, CommLink achieved near universal early enrollment in HIV care and ART initiation among all participants during Test and Start, including men and young adults, two groups with historically low ART coverage. To help achieve 90-90-90 by 2020, PEPFAR is supporting the national scale-up of CommLink in Eswatini and recommending peer-delivered linkage case management as a potential strategy for countries to achieve >90% early enrollment in care and ART initiation after HIV diagnosis (*6*).

**Summary**

**What is already known about this topic?**

Few (26%–37%) persons with human immunodeficiency virus (HIV) infection diagnosed in community settings in sub-Saharan Africa enroll early in care and initiate antiretroviral therapy (ART) when provided standard referral services, particularly men and young adults.

**What is added by this report?**

Among 651 persons diagnosed with HIV infection in community settings in Eswatini, 98% enrolled in care, and 83% initiated ART within a few days of receiving peer-delivered linkage case management services recommended by CDC and the World Health Organization. After expansion of ART eligibility for all persons with HIV infection, 96% initiated ART.

**What are the implications for public health practice?**

Providing recommended peer-delivered linkage case management services should be considered as a potential strategy for countries to help achieve >90% early enrollment in care and ART initiation after HIV diagnosis.

## Footnotes

* HIV RNA concentration below the threshold needed for detection on a viral load assay.

† Linkage case management is a time-limited, multicomponent, client-centered intervention focused on linking persons with HIV infection to medical care and antiretroviral therapy.

§ Cosponsors include the Health Resources and Services Administration, National Institutes of Health, and International Association of Providers of AIDS Care.

¶ Provision of ART for all persons living with HIV. http://www.who.int/hiv/pub/arv/arv-2016/en/.
